# 5-Pentyl-4-phenyl­sulfonyl-1*H*-pyrazol-3-ol

**DOI:** 10.1107/S1600536810019458

**Published:** 2010-05-29

**Authors:** Tara Shahani, Hoong-Kun Fun, R. Venkat Ragavan, V. Vijayakumar, S. Sarveswari

**Affiliations:** a X-ray Crystallography Unit, School of Physics, Universiti Sains Malaysia, 11800 USM, Penang, Malaysia; b Organic Chemistry Division, School of Advanced Sciences, VIT University, Vellore 632 014, India

## Abstract

In the title compound, C_14_H_18_N_2_O_3_S, the 1*H*-pyrazole ring is approximately planar, with a maximum deviation of 0.005 (1) Å. The dihedral angle formed between the 1*H*-pyrazole and phenyl rings is 79.09 (5)°. Pairs of inter­molecular N—H⋯O and O⋯H⋯N hydrogen bonds form dimers between neighboring mol­ecules, generating *R*_2_^2^(10) ring motifs. These dimers are further linked by intermolecular N—H⋯O and O—H⋯N hydrogen bonds into two-dimensional arrays parallel to the *ac* plane. The crystal structure is also stabilized by C—H⋯π inter­actions.

## Related literature

For background to the biological activity of 3-ethyl-4-methyl-1*H*-pyrazol-5-ol, see: Brogden (1986 ▶); Gursoy *et al.* (2000 ▶); Ragavan *et al.* (2009 ▶, 2010 ▶); Watanabe *et al.* (1984 ▶); Kawai *et al.* (1997 ▶); Wu *et al.* (2002 ▶). For related structures, see: Shahani *et al.* (2009 ▶, 2010**a* ▶,b* ▶). For hydrogen-bond motifs, see: Bernstein *et al.* (1995 ▶). For reference bond-length data, see: Allen *et al.* (1987 ▶). For the stability of the temperature controller used for the data collection, see: Cosier & Glazer (1986 ▶).
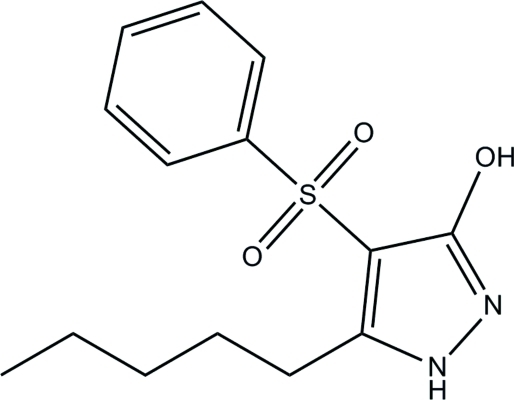


## Experimental

### 

#### Crystal data

C_14_H_18_N_2_O_3_S
*M* *_r_* = 294.36
Monoclinic, 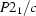

*a* = 10.3425 (3) Å
*b* = 11.2963 (3) Å
*c* = 12.8911 (3) Å
β = 107.419 (1)°
*V* = 1437.02 (7) Å^3^
*Z* = 4
Mo *K*α radiation
μ = 0.23 mm^−1^
*T* = 100 K
0.48 × 0.33 × 0.11 mm

#### Data collection

Bruker APEXII DUO CCD area-detector diffractometer
Absorption correction: multi-scan (*SADABS*; Bruker, 2009 ▶) *T*_min_ = 0.896, *T*_max_ = 0.976
28969 measured reflections
7810 independent reflections
6336 reflections with *I* > 2σ(*I*)
*R*_int_ = 0.035

#### Refinement

*R*[*F* ^2^ > 2σ(*F* ^2^)] = 0.037
*wR*(*F* ^2^) = 0.122
*S* = 1.14
7810 reflections
253 parameters
All H-atom parameters refined
Δρ_max_ = 0.73 e Å^−3^
Δρ_min_ = −0.41 e Å^−3^

### 

Data collection: *APEX2* (Bruker, 2009 ▶); cell refinement: *SAINT* (Bruker, 2009 ▶); data reduction: *SAINT*; program(s) used to solve structure: *SHELXTL* (Sheldrick, 2008 ▶); program(s) used to refine structure: *SHELXTL*; molecular graphics: *SHELXTL*; software used to prepare material for publication: *SHELXTL* and *PLATON* (Spek, 2009 ▶).

## Supplementary Material

Crystal structure: contains datablocks global, I. DOI: 10.1107/S1600536810019458/wn2386sup1.cif

Structure factors: contains datablocks I. DOI: 10.1107/S1600536810019458/wn2386Isup2.hkl

Additional supplementary materials:  crystallographic information; 3D view; checkCIF report

## Figures and Tables

**Table 1:** Hydrogen-bond geometry (Å, °) *Cg*1 is the centroid of the 1*H*-pyrazole ring (C7–C9/N1/N2).

*D*—H⋯*A*	*D*—H	H⋯*A*	*D*⋯*A*	*D*—H⋯*A*
O3—H1*O*3⋯N1^i^	0.945 (19)	1.79 (2)	2.7287 (10)	171.5 (17)
N2—H1*N*2⋯O2^ii^	0.880 (19)	1.959 (19)	2.7162 (10)	143.4 (17)
C12—H12*A*⋯*Cg*1^iii^	1.005 (16)	2.952 (16)	3.5692 (10)	120.5 (11)

Symmetry codes: (i) 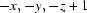; (ii) 
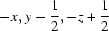
; (iii) 
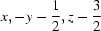
.

**Fig. 1.**
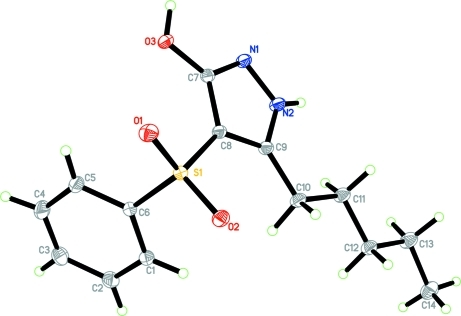
The molecular structure of the title compound, showing 30% probability displacement ellipsoids and the atom numbering scheme. Hydrogen atoms are shown as spheres of arbitrary radius.

**Fig. 2.**
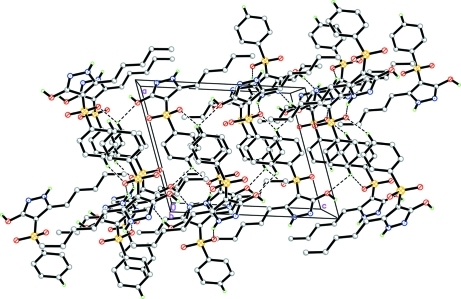
The crystal packing of the title compound, viewed along the b axis, showing 2D arrays parallel to the ac plane. Dashed lines indicate hydrogen bonds. H atoms not involved in the hydrogen bond interactions have been omitted for clarity.

## supplementary crystallographic information

### Comment

Pyrazolone derivatives have a broad spectrum of biological activities, being used as analgesic, antipyretic and anti-inflammatory therapeutical drugs (Brogden, 1986; Gursoy *et al.*, 2000). A class of new compounds with the pyrazolone unit has been synthesized and reported to possess antibacterial and antifungal activities (Ragavan *et al.*, 2009, 2010). A new pyrazolone derivative, edaravone (3-methyl-1-phenyl-2-pyrazoline-5-one), is being used as a drug in clinical practice for brain ischemia (Watanabe *et al.*, 1984; Kawai *et al.*, 1997) and the same has been found to be effective against myocardial ischemia (Wu *et al.*, 2002).

In the crystal structure (Fig. 1), the 1*H*-pyrazole ring (C1–C2/N1/N2) is approximately planar, with a maximum deviation of 0.005 (1) Å at atoms C9 and N2. The dihedral angle formed between the 1*H*-pyrazole and phenyl rings (C1—C6) is 79.09 (5)°. The bond lengths (Allen *et al.*,1987) and angles are within normal ranges and comparable to those in closely related crystal structures (Shahani *et al.*, 2009, 2010*a,b*).

In the crystal packing (Fig. 2), pairs of intermolecular O3—H1O3···N1, N2—H1N2···O2 hydrogen bonds form dimers between neighbouring molecules, generating *R*^2^_2_(8) ring motifs (Bernstein *et al.*, 1995). These dimers are linked into two-dimensional arrays parallel to the *ac* plane by intermolecular O3—H1O3···N1 and N2—H1N2···O2 hydrogen bonds (Table 1). The crystal structure is also stabilized by C12—H12A···*Cg*1 interactions (Table 1) involving the C7–C9/N1/N2 pyrazole ring.

### Experimental

The compound 5-pentyl-4-(phenylsulfonyl)-1*H*-pyrazol-3-ol was synthesized according to the procedure available in the literature (Ragavan *et al.*, 2009, 2010), which in turn was dissolved using a THF/water (1:1) mixture. Oxone (4 mmol) was then added and the solution was stirred at room temperature for 3 h. The reaction mixture was diluted with water (20 ml), and then was extracted with ethyl acetate (2 *x* 50 ml). The combined extract was washed with water (20 ml) and brine solution (10 ml). Crystallization was carried out using absolute ethanol.

### Refinement

All hydrogen atoms were located in a difference map and were refined freely [C—H = 0.910 (19) – 1.035 (18); N—H = 0.880 (18); O—H = 0.944 (19) Å].

### Crystal data

**Table:**

C_14_H_18_N_2_O_3_S	*F*(000) = 624
*M**_r_* = 294.36	*D*_x_ = 1.361 Mg m^−^^3^
Monoclinic, *P*2_1_/*c*	Mo *K*α radiation, λ = 0.71073 Å
Hall symbol: -P 2ybc	Cell parameters from 9972 reflections
*a* = 10.3425 (3) Å	θ = 2.5–37.9°
*b* = 11.2963 (3) Å	µ = 0.23 mm^−^^1^
*c* = 12.8911 (3) Å	*T* = 100 K
β = 107.419 (1)°	Plate, colourless
*V* = 1437.02 (7) Å^3^	0.48 × 0.33 × 0.11 mm
*Z* = 4	

### Data collection

**Table:**

Bruker APEXII DUO CCD area-detector diffractometer	7810 independent reflections
Radiation source: fine-focus sealed tube	6336 reflections with *I* > 2σ(*I*)
graphite	*R*_int_ = 0.035
φ and ω scans	θ_max_ = 38.1°, θ_min_ = 2.1°
Absorption correction: multi-scan (*SADABS*; Bruker, 2009)	*h* = −17→17
*T*_min_ = 0.896, *T*_max_ = 0.976	*k* = −19→19
28969 measured reflections	*l* = −22→22

### Refinement

**Table:**

Refinement on *F*^2^	Primary atom site location: structure-invariant direct methods
Least-squares matrix: full	Secondary atom site location: difference Fourier map
*R*[*F*^2^ > 2σ(*F*^2^)] = 0.037	Hydrogen site location: inferred from neighbouring sites
*wR*(*F*^2^) = 0.122	All H-atom parameters refined
*S* = 1.14	*w* = 1/[σ^2^(*F*_o_^2^) + (0.0676*P*)^2^ + 0.1076*P*] where *P* = (*F*_o_^2^ + 2*F*_c_^2^)/3
7810 reflections	(Δ/σ)_max_ < 0.001
253 parameters	Δρ_max_ = 0.73 e Å^−^^3^
0 restraints	Δρ_min_ = −0.41 e Å^−^^3^

### Special details

**Table:**

Experimental. The crystal was placed in the cold stream of an Oxford Cyrosystems Cobra open-flow nitrogen cryostat (Cosier & Glazer, 1986) operating at 100.0 (1) K.
Geometry. All esds (except the esd in the dihedral angle between two l.s. planes) are estimated using the full covariance matrix. The cell esds are taken into account individually in the estimation of esds in distances, angles and torsion angles; correlations between esds in cell parameters are only used when they are defined by crystal symmetry. An approximate (isotropic) treatment of cell esds is used for estimating esds involving l.s. planes.
Refinement. Refinement of *F*^2^ against ALL reflections. The weighted *R*-factor wR and goodness of fit *S* are based on *F*^2^, conventional *R*-factors *R* are based on *F*, with *F* set to zero for negative *F*^2^. The threshold expression of *F*^2^ > σ(*F*^2^) is used only for calculating *R*-factors(gt) etc. and is not relevant to the choice of reflections for refinement. *R*-factors based on *F*^2^ are statistically about twice as large as those based on *F*, and *R*- factors based on ALL data will be even larger.

### Fractional atomic coordinates and isotropic or equivalent isotropic displacement parameters (Å^2^)

**Table:**

	*x*	*y*	*z*	*U*_iso_*/*U*_eq_	
S1	0.252485 (19)	0.286811 (16)	0.369236 (14)	0.01071 (5)	
O1	0.28657 (7)	0.32531 (6)	0.48049 (5)	0.01659 (12)	
O2	0.21016 (7)	0.37440 (5)	0.28352 (5)	0.01507 (11)	
O3	0.16163 (6)	0.10556 (6)	0.53216 (5)	0.01448 (11)	
N1	−0.00782 (8)	0.03707 (6)	0.38100 (6)	0.01404 (12)	
N2	−0.04649 (8)	0.06672 (6)	0.27303 (6)	0.01496 (12)	
C1	0.40090 (9)	0.19978 (8)	0.24384 (7)	0.01491 (13)	
C2	0.50570 (9)	0.13165 (8)	0.22799 (7)	0.01884 (15)	
C3	0.59982 (10)	0.07844 (9)	0.31610 (8)	0.02123 (16)	
C4	0.59168 (10)	0.09408 (9)	0.42112 (8)	0.02094 (16)	
C5	0.48687 (9)	0.16047 (8)	0.43838 (7)	0.01639 (14)	
C6	0.39226 (8)	0.21172 (6)	0.34913 (6)	0.01197 (12)	
C7	0.09692 (8)	0.10767 (7)	0.42646 (6)	0.01194 (12)	
C8	0.12549 (8)	0.18213 (7)	0.34698 (6)	0.01188 (12)	
C9	0.02975 (8)	0.15129 (7)	0.24810 (6)	0.01305 (13)	
C10	0.00436 (9)	0.19276 (8)	0.13344 (7)	0.01604 (14)	
C11	−0.11449 (9)	0.12961 (8)	0.05249 (7)	0.01717 (15)	
C12	−0.13109 (9)	0.16318 (8)	−0.06542 (7)	0.01677 (14)	
C13	−0.24983 (11)	0.09921 (9)	−0.14480 (7)	0.02131 (17)	
C14	−0.25766 (12)	0.11849 (10)	−0.26364 (8)	0.02367 (18)	
H1A	0.3342 (16)	0.2363 (14)	0.1852 (12)	0.022 (3)*	
H2A	0.5111 (16)	0.1238 (14)	0.1558 (13)	0.025 (4)*	
H3A	0.6683 (17)	0.0309 (15)	0.3017 (13)	0.029 (4)*	
H4A	0.6624 (17)	0.0595 (14)	0.4792 (13)	0.031 (4)*	
H5A	0.4772 (17)	0.1722 (14)	0.5069 (13)	0.026 (4)*	
H10A	0.0893 (16)	0.1812 (13)	0.1116 (12)	0.021 (3)*	
H10B	−0.0097 (16)	0.2808 (13)	0.1271 (13)	0.022 (4)*	
H11A	−0.1995 (16)	0.1492 (13)	0.0666 (12)	0.021 (3)*	
H11B	−0.1003 (15)	0.0412 (13)	0.0581 (12)	0.020 (3)*	
H12A	−0.1437 (15)	0.2511 (14)	−0.0758 (12)	0.023 (3)*	
H12B	−0.0479 (18)	0.1422 (15)	−0.0867 (14)	0.031 (4)*	
H13A	−0.2409 (17)	0.0115 (17)	−0.1291 (13)	0.036 (5)*	
H13B	−0.3394 (18)	0.1256 (16)	−0.1315 (14)	0.034 (4)*	
H14A	−0.2628 (16)	0.2016 (13)	−0.2746 (13)	0.023 (4)*	
H14B	−0.3452 (19)	0.0787 (16)	−0.3118 (15)	0.037 (4)*	
H14C	−0.1814 (19)	0.0873 (16)	−0.2740 (15)	0.037 (4)*	
H1O3	0.1158 (19)	0.0555 (17)	0.5682 (14)	0.042 (5)*	
H1N2	−0.1111 (19)	0.0241 (16)	0.2289 (15)	0.039 (5)*	

### Atomic displacement parameters (Å^2^)

**Table:**

	*U*^11^	*U*^22^	*U*^33^	*U*^12^	*U*^13^	*U*^23^
S1	0.01150 (9)	0.01041 (8)	0.01036 (8)	−0.00003 (5)	0.00348 (6)	0.00008 (5)
O1	0.0196 (3)	0.0179 (3)	0.0126 (2)	−0.0023 (2)	0.0053 (2)	−0.0042 (2)
O2	0.0168 (3)	0.0120 (2)	0.0166 (2)	0.00187 (19)	0.0053 (2)	0.00367 (19)
O3	0.0152 (3)	0.0173 (2)	0.0106 (2)	−0.0011 (2)	0.0033 (2)	0.00243 (18)
N1	0.0157 (3)	0.0151 (3)	0.0112 (2)	−0.0019 (2)	0.0038 (2)	0.0015 (2)
N2	0.0165 (3)	0.0162 (3)	0.0115 (2)	−0.0045 (2)	0.0031 (2)	0.0013 (2)
C1	0.0139 (3)	0.0187 (3)	0.0123 (3)	0.0007 (2)	0.0043 (3)	0.0005 (2)
C2	0.0170 (4)	0.0240 (4)	0.0173 (3)	0.0017 (3)	0.0078 (3)	−0.0014 (3)
C3	0.0164 (4)	0.0244 (4)	0.0240 (4)	0.0054 (3)	0.0078 (3)	0.0012 (3)
C4	0.0168 (4)	0.0251 (4)	0.0200 (4)	0.0075 (3)	0.0043 (3)	0.0050 (3)
C5	0.0152 (3)	0.0198 (3)	0.0134 (3)	0.0034 (3)	0.0032 (3)	0.0031 (3)
C6	0.0108 (3)	0.0131 (3)	0.0119 (3)	0.0000 (2)	0.0032 (2)	0.0010 (2)
C7	0.0128 (3)	0.0122 (3)	0.0114 (3)	0.0008 (2)	0.0043 (2)	0.0006 (2)
C8	0.0123 (3)	0.0123 (3)	0.0112 (3)	−0.0010 (2)	0.0038 (2)	0.0008 (2)
C9	0.0138 (3)	0.0136 (3)	0.0119 (3)	−0.0021 (2)	0.0040 (2)	0.0005 (2)
C10	0.0171 (3)	0.0186 (3)	0.0112 (3)	−0.0048 (3)	0.0025 (3)	0.0019 (2)
C11	0.0181 (4)	0.0196 (3)	0.0126 (3)	−0.0052 (3)	0.0026 (3)	0.0008 (3)
C12	0.0183 (4)	0.0184 (3)	0.0127 (3)	−0.0032 (3)	0.0033 (3)	0.0012 (2)
C13	0.0239 (4)	0.0243 (4)	0.0132 (3)	−0.0059 (3)	0.0016 (3)	0.0004 (3)
C14	0.0290 (5)	0.0268 (4)	0.0134 (3)	0.0016 (4)	0.0037 (3)	0.0000 (3)

### Geometric parameters (Å, °)

**Table:**

S1—O1	1.4379 (6)	C5—H5A	0.928 (16)
S1—O2	1.4498 (6)	C7—C8	1.4233 (11)
S1—C8	1.7263 (8)	C8—C9	1.4039 (11)
S1—C6	1.7610 (8)	C9—C10	1.4969 (11)
O3—C7	1.3264 (10)	C10—C11	1.5300 (12)
O3—H1O3	0.944 (19)	C10—H10A	1.009 (15)
N1—C7	1.3315 (11)	C10—H10B	1.004 (15)
N1—N2	1.3697 (10)	C11—C12	1.5255 (12)
N2—C9	1.3377 (10)	C11—H11A	0.976 (16)
N2—H1N2	0.880 (18)	C11—H11B	1.009 (14)
C1—C6	1.3932 (11)	C12—C13	1.5248 (13)
C1—C2	1.3934 (12)	C12—H12A	1.005 (16)
C1—H1A	0.952 (15)	C12—H12B	1.006 (17)
C2—C3	1.3917 (14)	C13—C14	1.5254 (13)
C2—H2A	0.952 (16)	C13—H13A	1.010 (19)
C3—C4	1.3926 (14)	C13—H13B	1.035 (18)
C3—H3A	0.950 (17)	C14—H14A	0.949 (15)
C4—C5	1.3897 (13)	C14—H14B	1.034 (18)
C4—H4A	0.959 (16)	C14—H14C	0.910 (19)
C5—C6	1.3937 (11)		
			
O1—S1—O2	118.80 (4)	C7—C8—S1	126.64 (6)
O1—S1—C8	108.67 (4)	N2—C9—C8	105.38 (7)
O2—S1—C8	107.44 (4)	N2—C9—C10	121.26 (7)
O1—S1—C6	109.00 (4)	C8—C9—C10	133.35 (7)
O2—S1—C6	106.88 (4)	C9—C10—C11	113.28 (7)
C8—S1—C6	105.24 (4)	C9—C10—H10A	109.0 (8)
C7—O3—H1O3	110.0 (11)	C11—C10—H10A	109.8 (8)
C7—N1—N2	104.62 (6)	C9—C10—H10B	111.6 (9)
C9—N2—N1	113.89 (7)	C11—C10—H10B	109.8 (9)
C9—N2—H1N2	128.6 (12)	H10A—C10—H10B	102.9 (12)
N1—N2—H1N2	117.2 (12)	C12—C11—C10	113.05 (7)
C6—C1—C2	118.42 (8)	C12—C11—H11A	106.9 (9)
C6—C1—H1A	119.3 (9)	C10—C11—H11A	110.6 (9)
C2—C1—H1A	122.2 (9)	C12—C11—H11B	106.7 (8)
C3—C2—C1	120.24 (8)	C10—C11—H11B	110.1 (8)
C3—C2—H2A	121.7 (10)	H11A—C11—H11B	109.4 (12)
C1—C2—H2A	118.0 (10)	C13—C12—C11	112.25 (7)
C2—C3—C4	120.50 (9)	C13—C12—H12A	109.4 (9)
C2—C3—H3A	117.7 (10)	C11—C12—H12A	110.5 (8)
C4—C3—H3A	121.8 (10)	C13—C12—H12B	106.7 (10)
C5—C4—C3	120.07 (8)	C11—C12—H12B	111.3 (10)
C5—C4—H4A	122.9 (10)	H12A—C12—H12B	106.5 (13)
C3—C4—H4A	117.0 (10)	C12—C13—C14	113.34 (8)
C4—C5—C6	118.72 (8)	C12—C13—H13A	108.9 (10)
C4—C5—H5A	122.7 (10)	C14—C13—H13A	108.4 (9)
C6—C5—H5A	118.5 (10)	C12—C13—H13B	109.5 (10)
C1—C6—C5	122.01 (8)	C14—C13—H13B	110.1 (10)
C1—C6—S1	119.01 (6)	H13A—C13—H13B	106.4 (13)
C5—C6—S1	118.88 (6)	C13—C14—H14A	106.0 (9)
O3—C7—N1	122.36 (7)	C13—C14—H14B	108.3 (10)
O3—C7—C8	126.91 (7)	H14A—C14—H14B	109.9 (14)
N1—C7—C8	110.73 (7)	C13—C14—H14C	107.7 (11)
C9—C8—C7	105.37 (7)	H14A—C14—H14C	112.0 (15)
C9—C8—S1	127.99 (6)	H14B—C14—H14C	112.7 (15)
			
C7—N1—N2—C9	0.83 (10)	O3—C7—C8—S1	0.59 (12)
C6—C1—C2—C3	−0.77 (14)	N1—C7—C8—S1	−179.79 (6)
C1—C2—C3—C4	−0.99 (15)	O1—S1—C8—C9	155.48 (7)
C2—C3—C4—C5	1.82 (16)	O2—S1—C8—C9	25.76 (9)
C3—C4—C5—C6	−0.84 (14)	C6—S1—C8—C9	−87.89 (8)
C2—C1—C6—C5	1.76 (13)	O1—S1—C8—C7	−25.08 (8)
C2—C1—C6—S1	−174.63 (7)	O2—S1—C8—C7	−154.80 (7)
C4—C5—C6—C1	−0.96 (13)	C6—S1—C8—C7	91.55 (8)
C4—C5—C6—S1	175.44 (7)	N1—N2—C9—C8	−0.98 (10)
O1—S1—C6—C1	−159.60 (6)	N1—N2—C9—C10	178.47 (7)
O2—S1—C6—C1	−30.03 (7)	C7—C8—C9—N2	0.71 (9)
C8—S1—C6—C1	84.00 (7)	S1—C8—C9—N2	−179.75 (6)
O1—S1—C6—C5	23.90 (8)	C7—C8—C9—C10	−178.65 (9)
O2—S1—C6—C5	153.46 (7)	S1—C8—C9—C10	0.89 (14)
C8—S1—C6—C5	−92.50 (7)	N2—C9—C10—C11	0.26 (12)
N2—N1—C7—O3	179.32 (7)	C8—C9—C10—C11	179.54 (9)
N2—N1—C7—C8	−0.31 (9)	C9—C10—C11—C12	−174.25 (8)
O3—C7—C8—C9	−179.86 (8)	C10—C11—C12—C13	179.91 (8)
N1—C7—C8—C9	−0.25 (9)	C11—C12—C13—C14	−172.45 (8)

### Hydrogen-bond geometry (Å, °)

**Table:**

Cg1 is the centroid of the 1*H*-pyrazole ring (C7–C9/N1/N2).

**Table:**

*D*—H···*A*	*D*—H	H···*A*	*D*···*A*	*D*—H···*A*
O3—H1O3···N1^i^	0.945 (19)	1.79 (2)	2.7287 (10)	171.5 (17)
N2—H1N2···O2^ii^	0.880 (19)	1.959 (19)	2.7162 (10)	143.4 (17)
C12—H12A···Cg1^iii^	1.005 (16)	2.952 (16)	3.5692 (10)	120.5 (11)

Symmetry codes: (i) −*x*, −*y*, −*z*+1; (ii) −*x*, *y*−1/2, −*z*+1/2; (iii) *x*, −*y*−1/2, *z*−3/2.
